# Integration of an Independent Monitor Unit Check for High-Magnetic-Field MR-Guided Radiation Therapy System

**DOI:** 10.3389/fonc.2022.747825

**Published:** 2022-03-11

**Authors:** Jie Yang, Pengpeng Zhang, Neelam Tyagi, Paola Godoy Scripes, Ergys Subashi, Jiayi Liang, Dale Lovelock, James Mechalakos, Anyi Li, Seng B. Lim

**Affiliations:** Department of Medical Physics, Memorial Sloan-Kettering Cancer Center, New York, NY, United States

**Keywords:** MR-Linac, MU check, online adaptive plan, Elekta Unity, 1.5-T MR

## Abstract

**Purpose:**

Commercial independent monitor unit (IMU) check systems for high-magnetic-field MR-guided radiation therapy (RT) systems are lacking. We investigated the feasibility of adopting an existing treatment planning system (TPS) as an IMU check for online adaptive radiotherapy using 1.5-Tesla MR-Linac.

**Methods:**

The 7-MV flattening filter free (FFF) beam and multi-leaf collimator (MLC) models of a 1.5-T Elekta Unity MR-Linac within Monte Carlo-based Monaco TPS were used to generate an optimized beam model in Eclipse TPS. The MLC dosimetric leaf gap of the beam in Eclipse was determined by matching the dose distribution of Eclipse-generated intensity-modulated radiation therapy (IMRT) plans using the Analytical Anisotropic Algorithm (AAA) algorithm to Monaco plans. The plans were automatically adjusted for different source-to-axis distances (SADs) between the two systems. For IMU check, the treatment plans developed in Monaco were transferred to Eclipse to recalculate the dose using AAA. A plug-in within Eclipse was created to perform a 2D gamma analysis of the AAA and Monte Carlo dose distribution on a beam’s eye view parallel plane. Monaco dose distribution was shifted laterally by 2 mm during gamma analysis to account for the impact of magnetic field on electron trajectories. Eclipse doses for posterior beams were corrected for both the Unity couch and the posterior MR coil attenuation. Thirteen patients, each with 4–5 fractions for a variety of tumor sites (pancreas, rectum, and prostate), were tested.

**Results:**

After thorough commissioning, the method was implemented as part of the standard clinical workflow. A total of 62 online plans, each with approximately 15 beams, were evaluated. The average per-beam gamma (3%/3 mm) pass rate for plans was 97.9% (range, 95.9% to 98.8%). The average pass rate per beam for all 932 beams used in these plans was 97.9% ± 1.9%, with the lowest per-beam gamma pass rate at 88.4%. The time for the process was within 3.2 ± 0.9 min.

**Conclusion:**

The use of a second planning system provides an efficient way to perform IMU checks with clinically acceptable accuracy for online adaptive plans on Unity MR-Linac. This is essential for meeting the safety requirements for second checks as outlined in American Association of Physicists in Medicine Task Group (AAPM TG) reports 114 and 219.

## Introduction

MRI provides a huge advantage for target and organ at risk (OAR) delineation because of the superior soft-tissue contrast (1). Recent developments in on-board MRI coupled to the teleradiotherapy unit are enabling online MR-guided adaptive radiotherapy planning (2–4). The capability of evaluating daily geometric and anatomical changes along with real-time imaging of tumor position during beam delivery makes on-board MR superior to other imaging modalities for online treatment plan adaptation (1). The clinical introduction of such MR-guided radiotherapy (MRgRT) systems using hybrid MR-Linac systems have also prompted considerations of the potential impact of the static magnetic field on biological responses to radiation (5, 6). Early studies have reported initial results suggesting that the biological response to radiation in the presence of a static magnetic field (B-field) may be modestly different when compared to conventional radiotherapy in a zero B-field environment. While the mechanisms of interaction in the presence of a static magnetic field are still an active field of research, the calculation and validation of physical dose will be critical in isolating any biological effects due to treatment in a high-field MR-Linac system (4–6).

One such high-field hybrid MR-Linac system called Elekta Unity (Elekta AB, Stockholm, Sweden) comprises a 1.5-T MRI system (Philips, Best, The Netherlands) and a 7-MV Linac (Elekta AB, Stockholm, Sweden). The system uses the Monaco Treatment Planning System (TPS) (Elekta AB, Stockholm, Sweden), employing the GPUMCD Monte Carlo algorithm to account for the effect of 1.5-Tesla magnetic field during particle transport (7). During an online adaptive treatment session, a pretreatment MR image set is taken and fused with a reference CT or MR image to account for the daily setup shifts. While the patient is still in the treatment position, the reference contours and plan, generated based on a CT set from simulation or an MR image from a previous fraction, are adapted to the anatomy of the day, and the new plan is used for treatment delivery. As of the time of this study, a commercial solution for the independent check for online adaptive plans is not yet available with this hybrid system. Commercially available independent dose calculation algorithm lacks the B-field correction at the time of this study, rendering worse dosimetric agreement than conventional radiation therapy (RT) and requiring gamma tolerance to be widened as much as 5%/5 mm (8), especially in the presence of heterogeneity where the effect of electron return effect due to B-field is large. Appropriate quality assurance (QA) criteria, such as gamma pass rate, have not yet been established for MR-guided radiotherapy. To ensure the safety of the delivery (9), several in-house solutions were developed by different institutions to perform an independent monitor unit (IMU) QA (4, 8, 10–13). Most of them perform the IMU check by using dose engines from clinical TPSs or commercial QA software, such as Raystation Collapsed Cone (11), Oncentra Collapsed Cone (12), Mobius 3D (13), and RadCalc (8). These groups have also used gamma pass rates ranging from 3%/3 mm to 5%/5 mm. Since our institution already uses the widely available Varian Eclipse TPS, we sought to investigate the use and ease of integration of the Eclipse TPS as a fast and independent IMU check for the QA of online adaptive re-planning of Monaco plans on the Elekta MR-Linac system. The multi-leaf collimator (MLC) model of Elekta Agility Linac is available in Eclipse and provides an avenue to develop an IMU check for online adaptive plans from Monaco TPS.

## Methods and Materials

The IMU check is just one component of our patient-specific QA (PSQA) program in MR-guided RT (14). The IMU check used the same MUs from the Monaco plans as the input to the independent dose calculation and performed the QA check by comparing the dose distribution calculated with the two algorithms. An independent beam model in Eclipse that is equivalent to the beam model used in Monaco was generated first. The IMU workflow was streamlined by performing a DICOM transfer of the plan from Monaco to Eclipse. An Eclipse plugin was developed to perform the gamma analysis using the Eclipse Scripting Application Program Interface (ESAPI) software library provided by Varian and to generate a report for the QA record. In the following sections, we describe our commissioning and implementation in detail.

### Accounting for B-Field Effects in Eclipse

Unity is a 7-MV FFF Linac guided by a 1.5-T MRI system. To minimize the effect of the magnetic field on the dose distribution, the Linac components are placed above the MR-cryostat, resulting in a source-to-axis distance (SAD) of 143.5 cm, an effective dose rate of 450 MU/min, and a projected MLC leaf width of 7 mm. The magnetic field affects the electron trajectory inside the patient geometry, forcing them to move in a helical path. The net effect is approximately a lateral shift of the transverse profile (15, 16). The effect of the B-field in the IMU program using Eclipse TPS was approximated by performing a lateral shift in dose distributions that are calculated without the effect of the B-field. The overall advantage is that the dose calculation can be performed relatively quickly (within 2 min) in the 3D heterogeneous patient geometry.

### MR Images for Eclipse Dose Calculation

A new adaptive plan is generated in Monaco for every fraction using synthetic CTs generated on the fly by bulk electron density assignment derived from the reference planning CT. During the first treatment fraction, planning CT is used as a reference, and average bulk electron density within a structure is propagated from CT to the MR. During subsequent fractions, the average bulk electron density is propagated from MR to MR. Within each structure, the average electron density information is stored in DICOM headers and gets propagated to Eclipse.

### Beam Model and Multi-Leaf Collimator Model in Eclipse™ Treatment Planning System

In this study, we utilized Analytical Anisotropic Algorithm (AAA) (17) from Eclipse TPS as the independent dose calculation algorithm for secondary check QA of online adaptive plans. The major difference between Elekta Unity and this Elekta Versa in Eclipse is the SAD, which is fixed at 100 cm in Eclipse. The source to MLC distance is different in the two systems as well. But in Eclipse, this distance is not relevant to dose calculation, as the beamlets are projected at the isocenter by beam fan lines (17). Because of the source-to-surface distance (SSD) limitation of the Eclipse TPS for beam model data, the percent depth dose (PDD) and dose profile were generated within the Monaco TPS at an SSD of 100 cm so that they could be used as inputs to the Eclipse TPS. To achieve this, we generated a virtual phantom in Monaco at an SSD of 100 cm. The field size, defined at an SSD of 100 cm, varied from 1 × 1 to 40 × 40 cm^2^. Output factors were measured on Elekta Unity MR-Linac at a depth of 10 cm and a SAD of 143.5 cm with PTW (Freiburg, Germany) diamond detector and PTW Semi-flex 3D ion chamber for 1 × 1 to 3 × 3 cm^2^ and 2 × 2 to 57 × 22 cm^2^, respectively, in water. The measurements of the diamond detector were normalized to the ion chamber measurements at 3 × 3 cm^2^. In terms of absolute calibration, both Eclipse and Monaco TPS were set to have 1 cGy/MU at an SSD of 138.5 cm and a depth of 5.0 cm matching our Unity MR-Linac system. The 6-MV FFF Elekta Versa HD machine model was used as a starting point. The beam configuration of Eclipse TPS was then performed to generate an optimized beam model based on the 7-MV FFF input data. A variety of intensity-modulated RT (IMRT) plans generated in Monaco were used to determine the dosimetric leaf gap of the MLC model in Eclipse. The dosimetric leaf gap was adjusted in Eclipse so that the dose distribution for the above IMRT plans was best matched between Eclipse and Monaco by minimizing the dose difference.

The commissioning measurements for MR-Linac were done for dosimetry of MLC delivery, radiation output, beam profile constancy, and patient-specific QA for the first 50 treatments. Measurements were made in water or water-equivalent plastic using ion chambers of various sizes, an ion chamber array, MR-compatible 2D/3D diode array, portal imager, MRI, and radiochromic film. Results from end-to-end QA using anthropomorphic phantoms were included as a reference for baseline comparisons. The details of our commissioning and longitudinal QA performance of the Unity system are described in a separate published paper (18).

### Independent Monitor Unit Check Workflow

**Figure 1:**
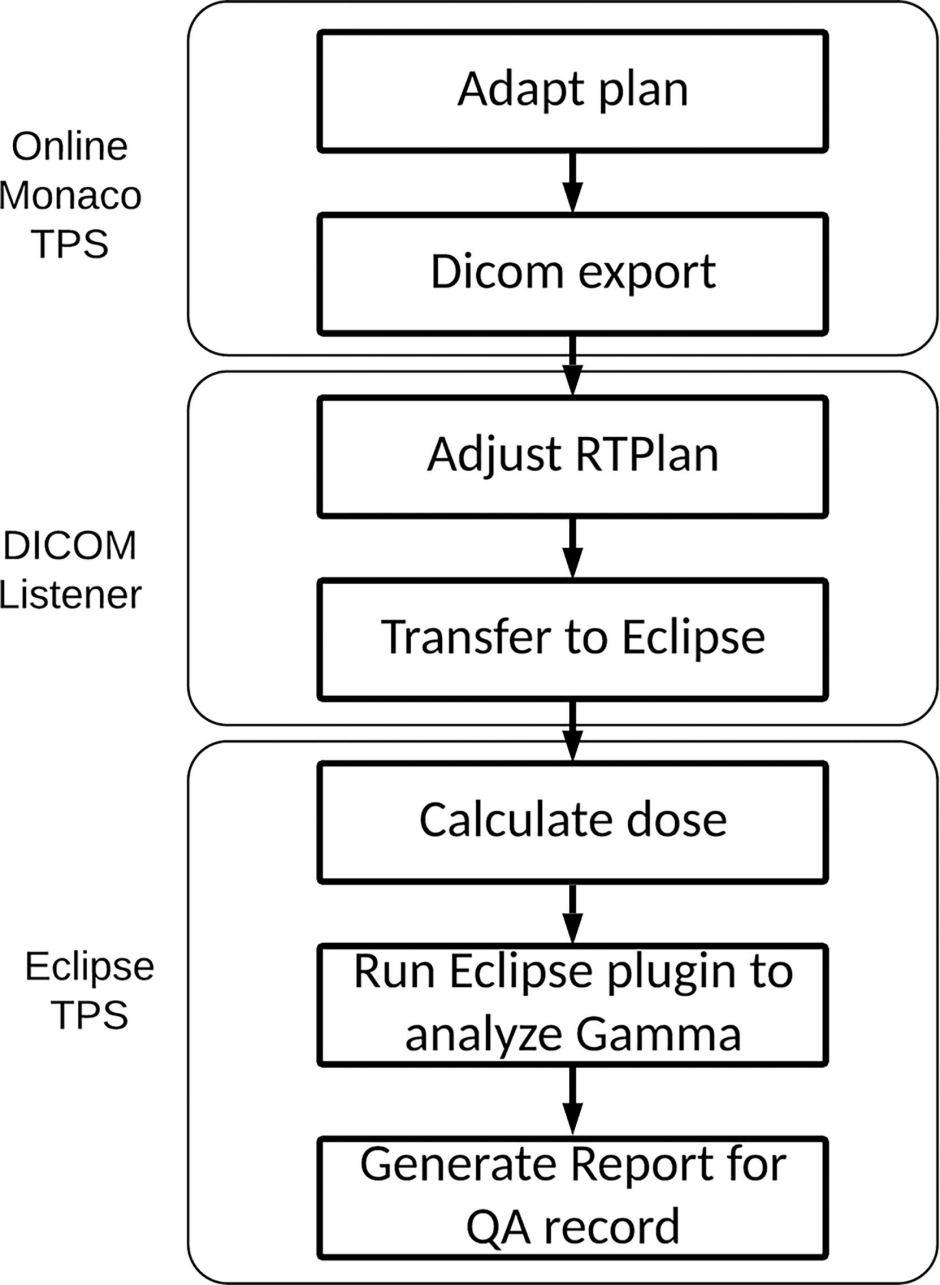
IMU workflow. IMU, independent monitor unit.

The workflow for our IMU program is illustrated in **Figure 1**. Once an online plan is generated in Monaco using the GPUMCD dose calculation algorithm and approved by the physician, the plan along with the MR images and structures is exported in DICOM format to our in-house DICOM listener. The listener is a service program on a server and processes the incoming DICOM files as needed and sends them to the Eclipse Daemon service on the Eclipse server. A physicist opens the imported plan in Eclipse and calculates the dose with fixed MUs on the online MR image. The average electron density of each structure is also exported with the plan file from Monaco to allow dose calculation in a patient heterogeneous geometry on the MR images. Once the dose calculation is completed, an in-house developed Eclipse ESAPI plugin program is run to perform a gamma comparison between the Monaco dose and Eclipse dose. The results from beam-by-beam gamma comparison can be printed to a patient e-folder for record keeping.

### DICOM Modification

The DICOM RT Plan exported from Monaco required modifications to accommodate the machine definition requirement of Eclipse and to facilitate proper dose calculation. This was incorporated in our DICOM listener service program to expedite the workflow. The following modifications are performed for every plan.

**Figure 2:**
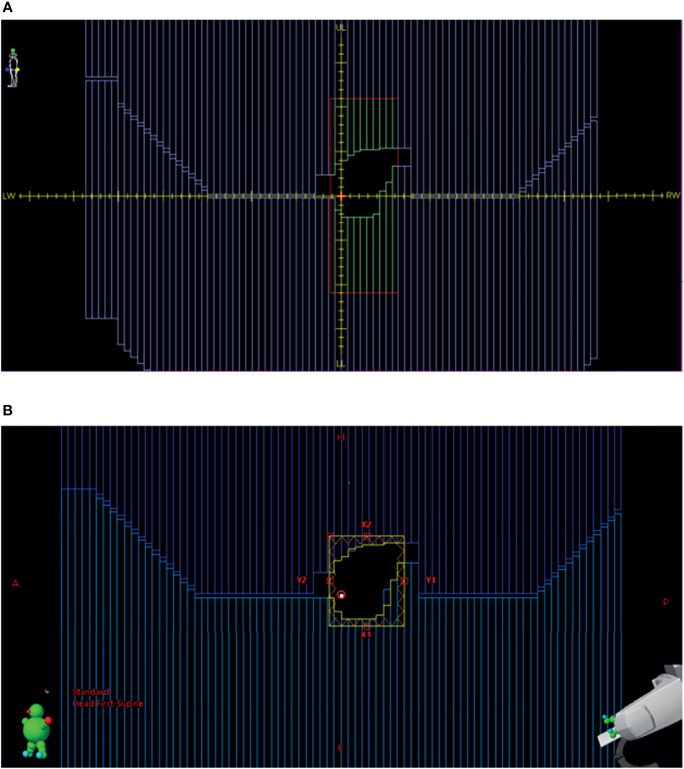
**(A)** BEV for a beam in Monaco. **(B)** BEV for the same beam imported into Eclipse from the converted RT plan. BEV, beam’s eye view; RT, radiation therapy.

1) To circumvent the 100-cm SAD limitation of Eclipse TPS, each beam was changed from SAD technique to extended SSD technique to retain the correct source distance. The collimator and MLC leaf positions specified by Monaco TPS are defined at a SAD of 143.5 cm. These values are scaled to a SAD of 100.0 cm, as required by Eclipse TPS, by a factor of 1.435 so that the projected MLC leaf positions are intact in the original Monaco beam’s eye view (BEV) plane.
2) Beam energy is nominally changed to 6 from 7 MV. This step can be eliminated if the beam is labeled as 7 MV in Eclipse.
3) The beam DICOM coordinate system is different between Monaco and Eclipse (**Figures 2A, B**). In Monaco, the MLC orientation is fixed as shown in **Figure 2A**. Although the diaphragm labels can be configured in the Monaco TPS, we use this convention, allowing test plans to be shared with the vendor and among collaborating institutions within the Elekta MR-Linac consortium. The x-axis is along the LeftWidth-RightWidth (LW-RW) direction indicated in the figure. The collimator angle is always 0° in the RT Plan exported from Monaco. There are no moving Y jaws in Monaco. Instead, the jaw size is always 22 cm in the superior–inferior direction (indicated by UpperLength (UL) and LowerLength (LL) in **Figure 2A**). Hence, there is no Y jaw position in a Monaco RT Plan file. The MLC leaf positions are stored in the DICOM tag MLCY. For Eclipse, the convention is different. The MLC leaf positions are stored in the DICOM tag MLCX. To make the RT Plan compatible with the Eclipse DICOM conformance requirement, we change the tag from MLCY to MLCX. The collimator angle is changed to 90° from 0°. The x- and y-axes are interchanged. New X jaw positions are created for Eclipse by taking the minimum and maximum leaf positions and adding a 5-mm margin. One can also use the fixed ±7.67 cm (projected from ±11 cm) as the X jaws. The dose difference is minimal in Eclipse between these two options.

Once the RT Plan file is converted, it is automatically sent to Eclipse DB Daemon where the DB Daemon service processes the received files and stores the CT/Structures/Plan in the Eclipse ARIA database. The Monaco beam RT Dose files are saved in a location for gamma analysis. They are not sent to Eclipse to save the processing time.

### Dose Corrections for Eclipse Dose

**Figure 3:**
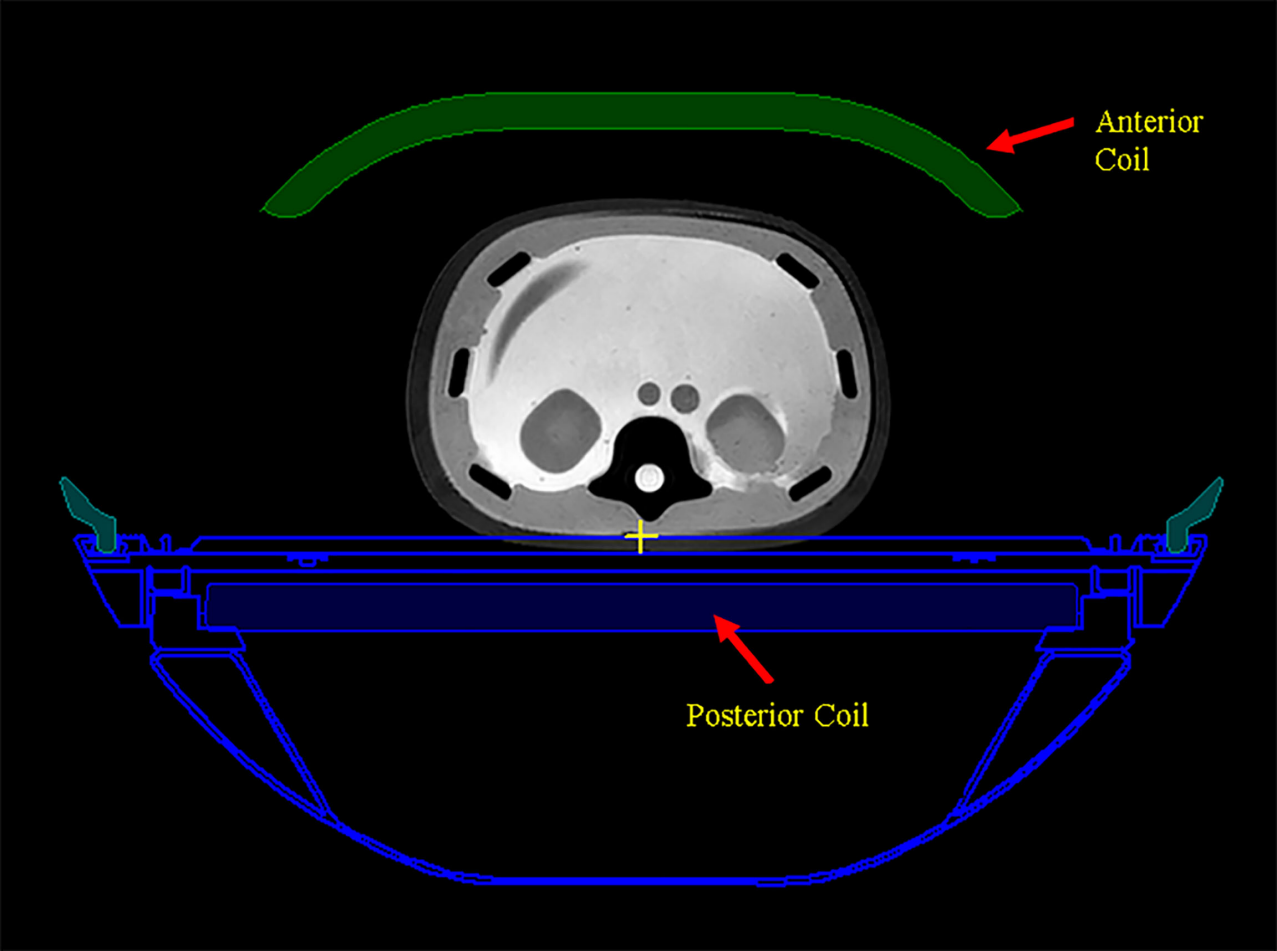
The scanned couch components are shown as blue contours. Anterior and posterior MR radio frequency (RF) coils are shown as green color-filled structure and blue color-filled structure, respectively.

The scanned couch contours with assigned electron density are also automatically transferred to Eclipse as part of the structure set. The couch is shown in an axial image slice in Monaco in **Figure 3**. The receiving RF coils in MR-Linac, also shown in **Figure 3**, are not included in the structures and are ignored by the dose calculation in Eclipse. The ionization chamber measurements by the Netherland group (19) showed that the attenuation effect is 0.4% for the anterior RF coil and 2.2% for the posterior RF coil. A uniform 2.0% attenuation is used in the Monaco dose calculation to account for this. For our gamma analysis, we applied 2.2% correction to the Eclipse dose for the posterior RF coil and ignored the anterior coil.

### Gamma Analysis for Dose Plane Comparison

**Figure 4:**
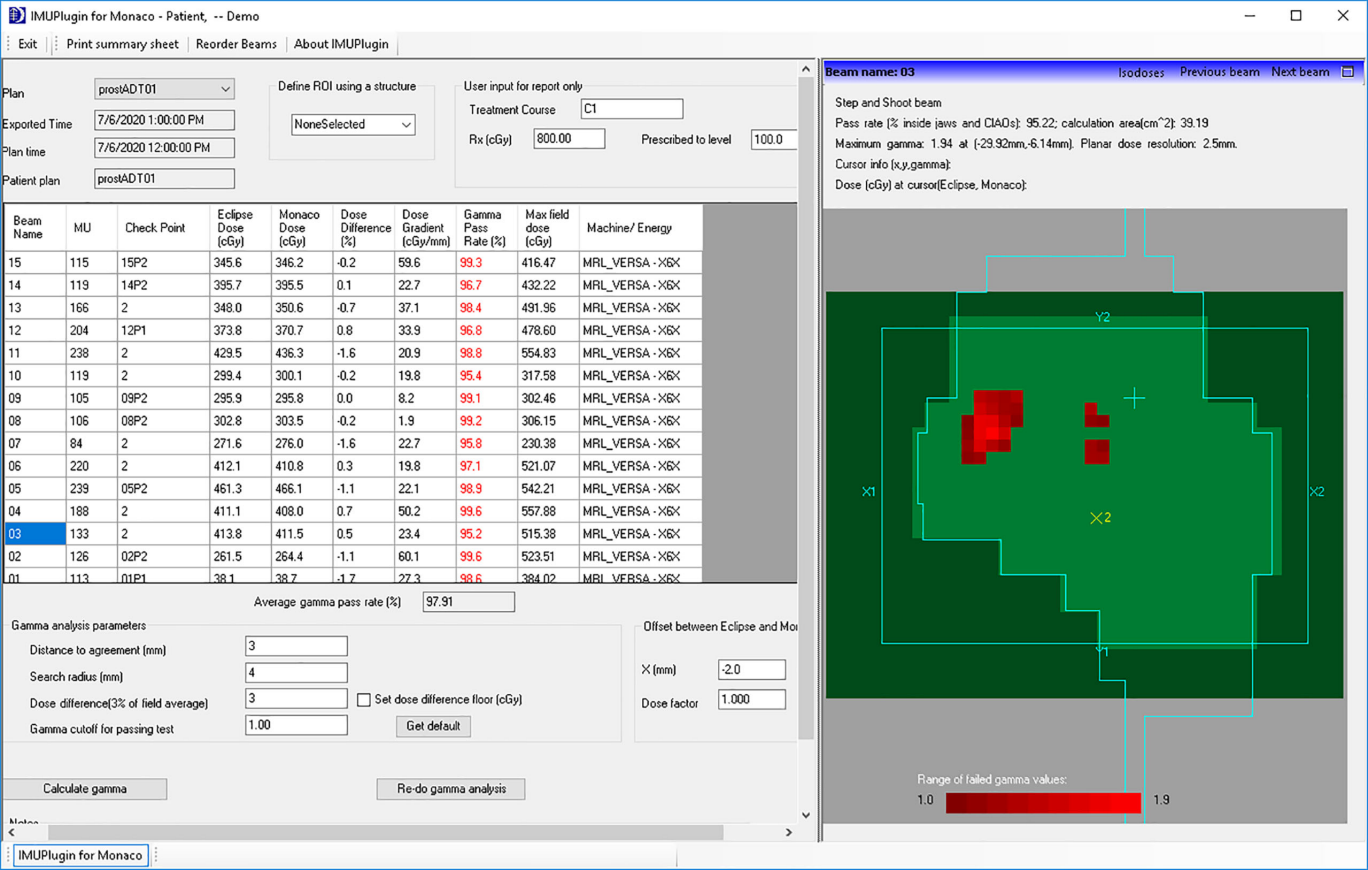
In-house developed Eclipse plugin for per-beam gamma analysis and point dose comparison.

The gamma analysis (20) is performed using our in-house developed Eclipse scripting plugin accessed *via* ESAPI. The interface of the plugin for an example patient is shown in **Figure 4**. For each beam, the plugin extracts the planar dose at a default plane from the Eclipse dose matrix as well as the planar dose at the same plane from the exported Monaco dose matrix. The default plane is the BEV parallel plane perpendicular to the beam axis through the default reference point in the plan. The planar dose distribution from Monaco is shifted 2 mm along the Eclipse BEV-Y direction before being used for comparison with the Eclipse distribution to account for the B-field effect. This BEV-Y axis is always perpendicular to the B-field and the beam central axis. The complete irradiated area outline (CIAO) of the beam is used to define the region of interest to calculate the gamma pass rate. The gamma passing rates were studied using two sets of gamma parameters: 3% of the maximum dose and the distance to agreement of 3 mm, and the same 3% and 2 mm as the distance to the agreement.

As part of preclinical commissioning of the system, a total of 94 beams, of which 18 were purposely mismatched to assess the IMU’s sensitivity to identify incorrect plan dose, were analyzed. They were also delivered with ArcCheck-MR (Sun Nuclear Corp., Melbourne, FL, USA). Gamma analysis of the measurements was first generated by comparing them with the dose calculation from Monaco. They were then correlated with the results from IMU with contingency analysis (14). The passing criteria were set at 95%.

Thirteen patients treated on the MR-Linac were analyzed for this study. Patient details including anatomical sites, dose fractionation, and number of beams are shown in **Table 1**. A total of 62 adapted plans (932 beams) were studied.

**Table 1:** Plan characteristics.

	Anatomical sites	# of patient	Dose fractionation	# of beams
1.	Pancreas	7	10 Gy × 5 fx	15
2.	Rectum boost	2	2 Gy × 4 fx	14, 15
3.	Pararectal lymph node	1	10 GY × 6 fx	15
4.	Uterus/rectum	1	8 Gy × 6 fx	16
5.	Prostate with DIL boost	1	8 Gy × 5 fx	15
6.	Prostate bed	1	7 Gy × 5 fx	15

DIL, dominant intraprostatic lesion.

## Results

### Beam Model and Multi-Leaf Collimator Model in Eclipse™ Treatment Planning System

**Figure 5:**
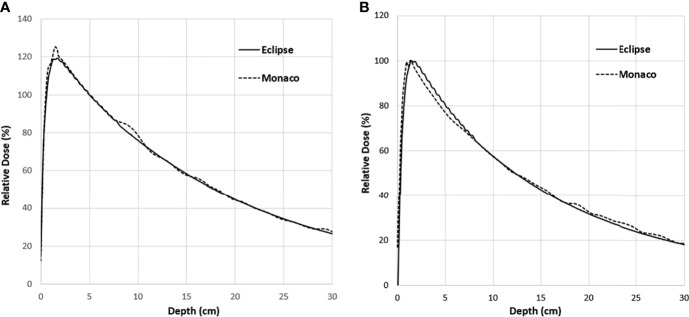
PDD comparison between Monaco and Eclipse for 10 × 10 cm^2^ field **(A)** and 2 × 2 cm^2^ field **(B)**. Monaco dose was calculated with the B-field. The difference around the buildup region is primarily due to the B-field. PDD, percent depth dose.

**Figure 6:**
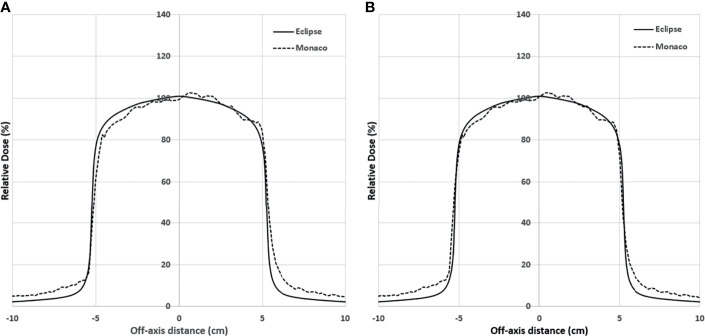
**(A)** Lateral profile comparison between Monaco and Eclipse dose calculation at depth 5 cm for 10 × 10 cm^2^. Monaco dose was calculated with the B-field. Compared to the Monaco TPS, Eclipse tends to underestimate the out-of-the-field dose due to the deficiency of its photon scatter modeling. **(B)** Comparison of the same distributions but with the Monaco distribution shifted by 2 mm. TPS, treatment planning system.

**Figure 7:**
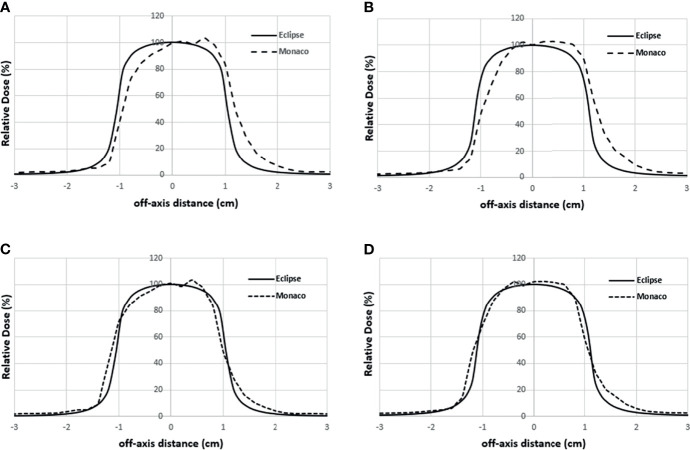
Lateral profile comparisons between Monaco and Eclipse dose calculation for 2 × 2 cm^2^ field size at depth 5 cm **(A)** and depth 10 cm **(B)**. Monaco dose was calculated with the B-field. **(C, D)** The comparisons of the same Eclipse profile and the Monaco profile shifted by 2 mm in panels A and B, respectively.

**Figure 5A** shows the comparison of the final Eclipse calculated PDD with the Monaco calculated PDD with the B-field for 10 × 10 cm^2^ field using 1% statistical uncertainty and 1-mm dose grid. The average PDD difference between Monaco and Eclipse is 0.8% at between 3.0 and 20.0 cm of depth. The comparison of the final Eclipse profile at a depth of 5 cm and an SSD of 100 cm for 10 × 10 cm^2^ field with the Monaco profile with the B-field is shown in **Figure 6A**. Because of the Lorentz force, the electrons are deflected preferentially in the +X direction, resulting in an asymmetric penumbra on either side of the field. The optimal lateral shift needed to make the two profiles in the best agreement was 2 mm, as shown in **Figure 6B**. **Figure 5B** shows a similar comparison of calculated PDD between Eclipse and Monaco for a 2 × 2 cm^2^ field. The comparisons of the profiles for 2 × 2 cm^2^ field are shown in **Figure 7A** for a depth of 5 cm and in **Figure 7B** for a depth of 10 cm. **Figures 7C, D** show the profile comparisons when the Monaco distribution is shifted by 2 mm. Both show good agreement. Therefore, the same 2-mm shift was applied to all depths, as there was no observed strong dependence of this shift value on the depth.

### Gamma Analysis of Clinical Plans

**Figure 8:**
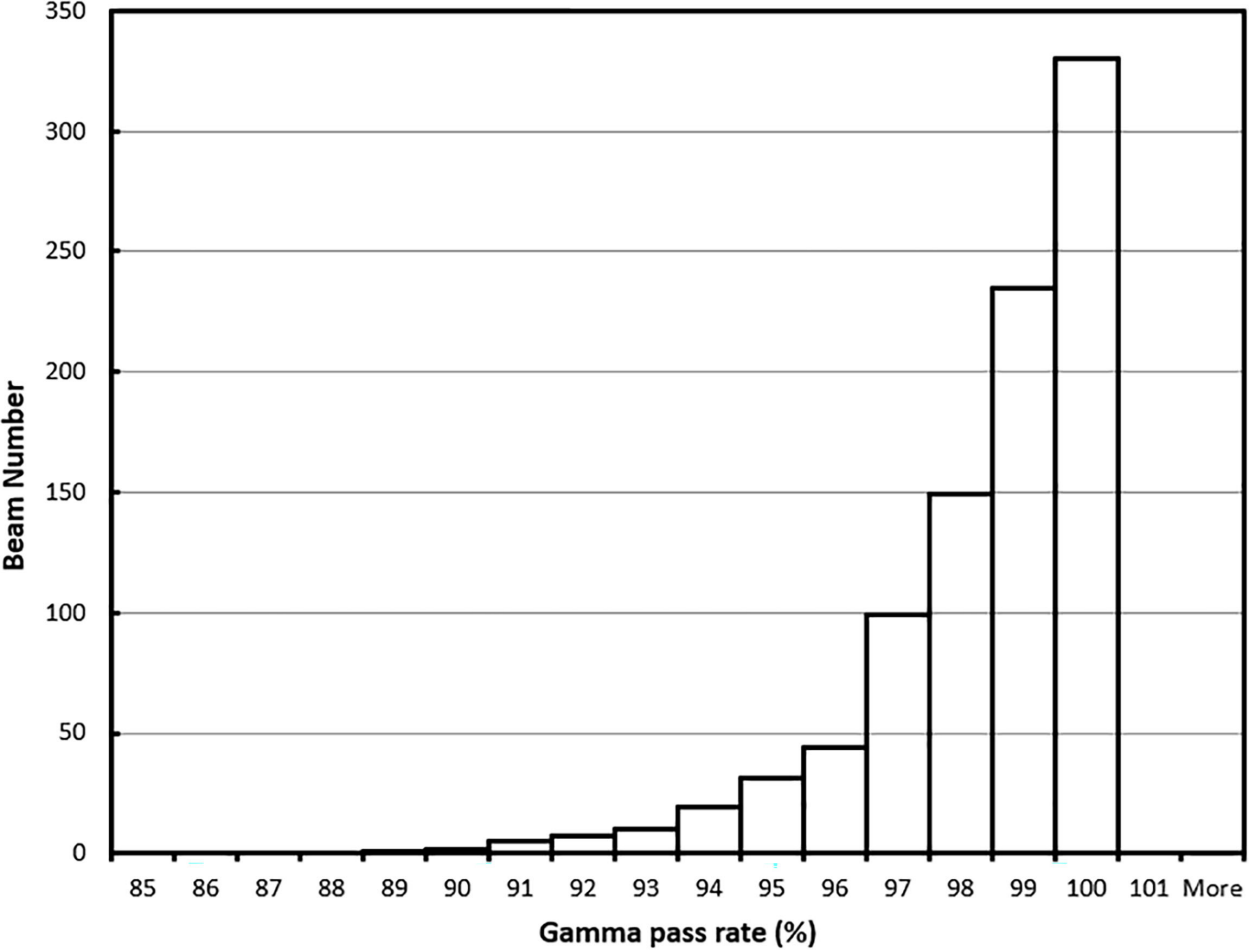
Gamma (3%/3 mm) pass rate histogram for all beams.

For the 62 online adapted plans, the average per-beam gamma pass rate using 3%/3 mm criteria was 97.9%. The maximum and minimum average plan pass rates were 98.8% and 95.9%, respectively. The average pass rate for all 932 beams used in these plans was 97.9% ± 1.9%. The maximum and minimum per beam pass rates were 100% and 88.4%, respectively. The histogram of the per-beam pass rate for all beams is shown in **Figure 8**.

**Figure 9:**
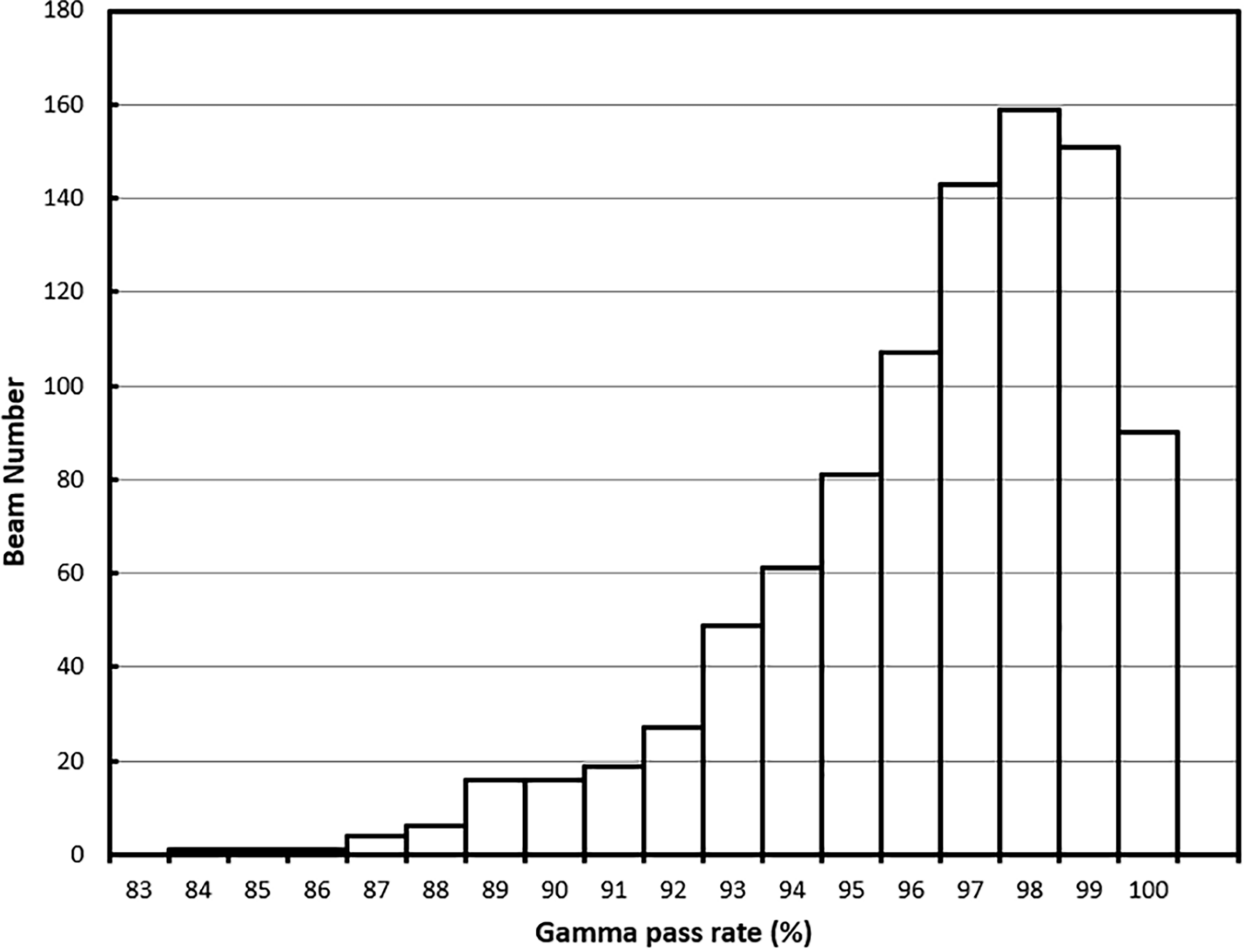
Gamma (3%/2 mm) pass rate histogram for all beams.

For all the 62 plans, the average per-beam gamma pass rate using 3%/2-mm criteria was 96.0%. The maximum and minimum average plan pass rates were 98.1% and 92.4%, respectively. The average pass rate for all beams was 96.0% ± 2.8%. The maximum and minimum per beam pass rates were 100% and 83.3%, respectively. The histogram of the per-beam pass rate for all beams is shown in **Figure 9**.

**Figure 10:**
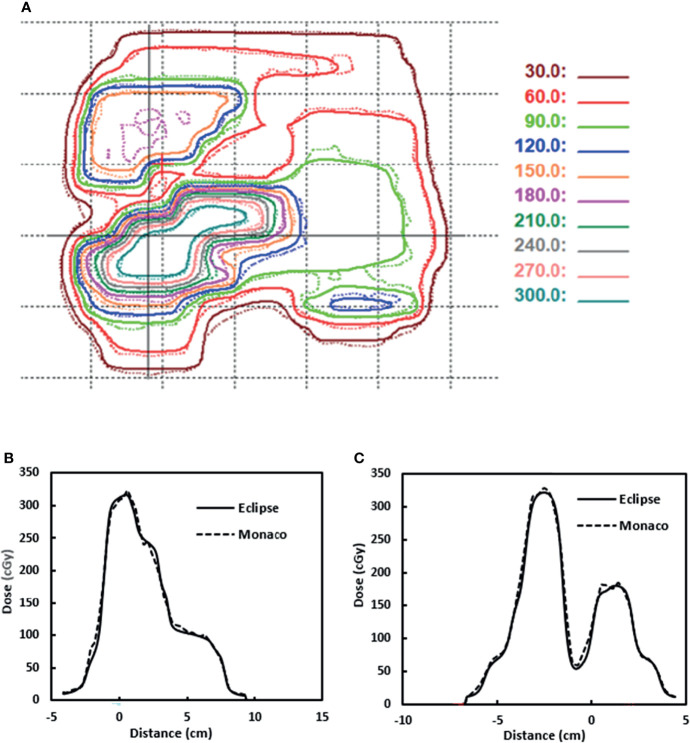
**(A)** Dose distributions used for gamma analysis for one beam. Eclipse dose distribution is in solid lines. Monaco dose distribution is in dashed lines. **(B)** Dose profile comparison for a horizontal line indicated by the solid horizontal line in panel **(A)**. **(C)** Dose profile comparison for a vertical line indicated by the solid vertical line in panel **(A)**. Distance is from the projected isocenter [the cross in panel **(A)**].

**Figure 10A** shows one example of the dose distributions on a BEV plane for gamma analysis. The Eclipse dose distribution is in solid lines. The Monaco dose distribution is in dashed lines and is already shifted by 2 mm. **Figures 10B, C** show the line profile comparisons for a horizontal line and a vertical line (indicated by two solid lines in **Figure 10A**), respectively. Both figures show good agreement between Eclipse and Monaco.

**Figure 11:**
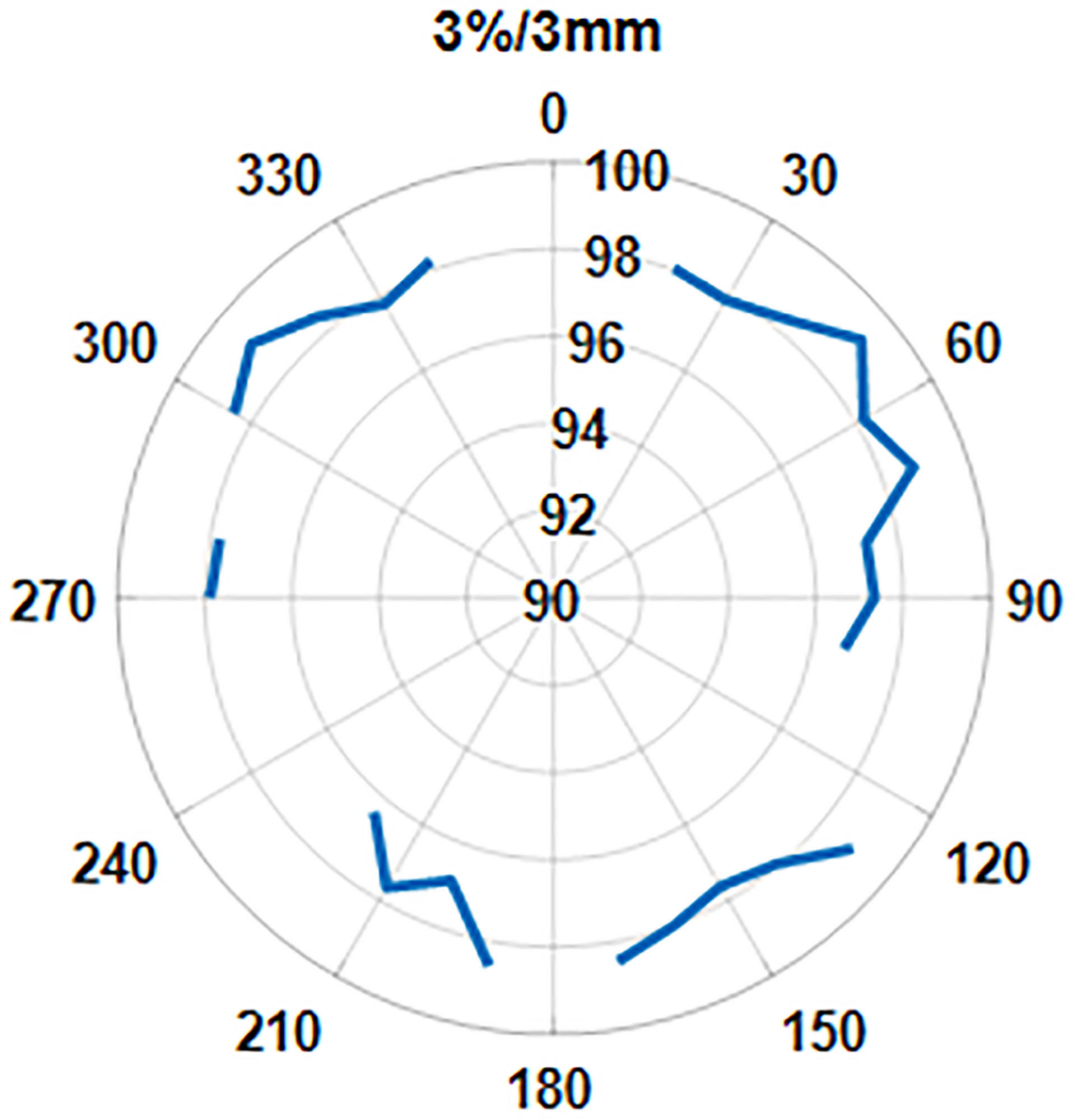
The pass rate distribution as a function of gantry angle.

We also looked at the pass rate vs. the beam angle. **Figure 11** shows that the gamma pass rate (3%/3 mm) for all the beam angles is >96% and shows no correlation of pass rate values with the beam angle.

**Figure 12:**
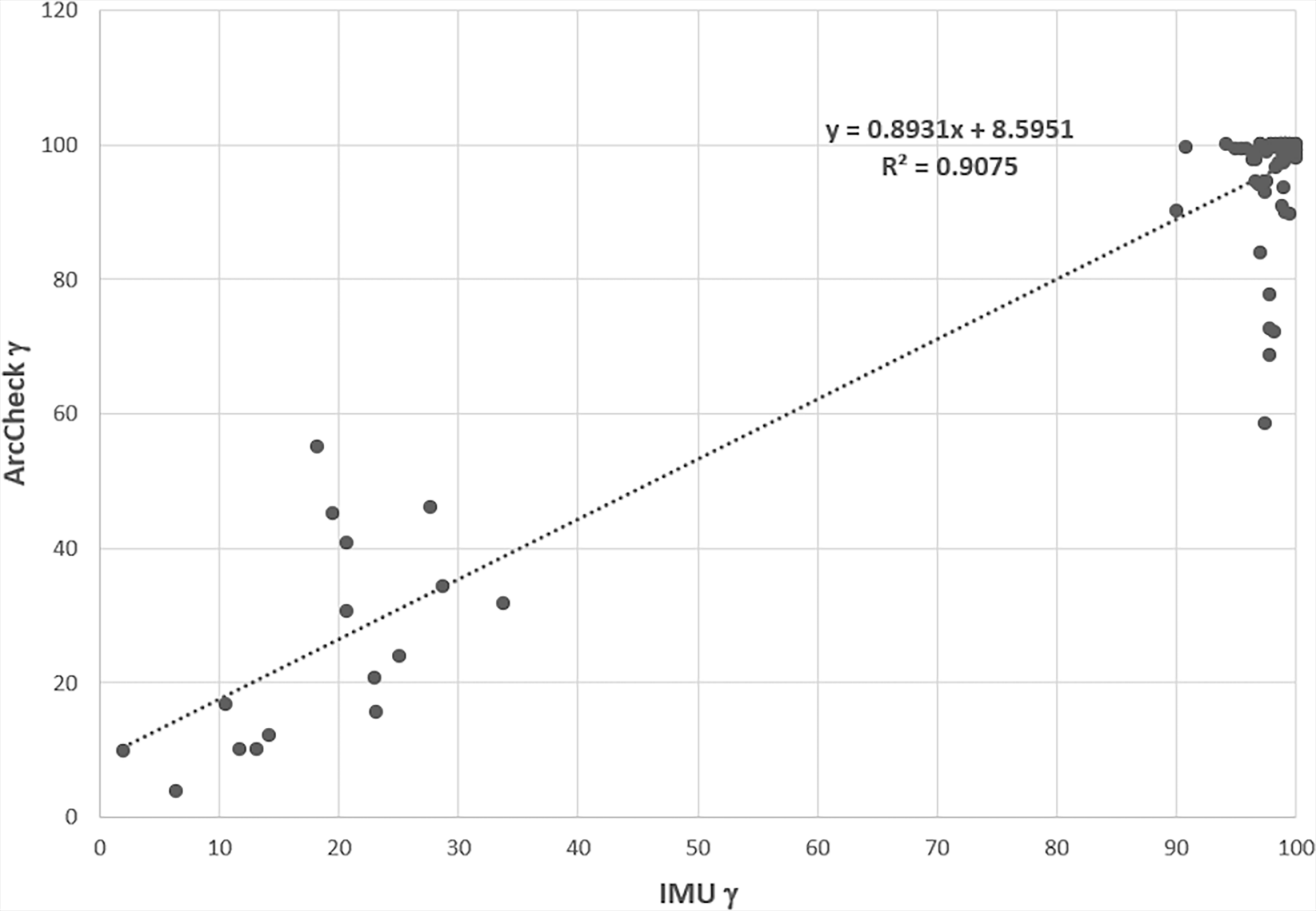
ArcCheck gamma pass rate vs. the IMU gamma pass rate for some of beams. IMU, independent monitor unit.

The contingency correlation of the preclinical ArcCheck and IMU was found to be 0.56 with a p-value of <0.001. **Figure 12** shows the correlation of the ArcCHECK gamma pass rates vs. the IMU pass rates. The slope and R^2^ of the best fit line were found to be 0.8931 and 0.9075, respectively. Both the contingency and linear fit showed a significant association between the measurements and calculation analysis. Some outliners were observed, which could be attributed to the modeling deficiency of the couch. As a result of this, an effort was made to minimize the use of gantry angle ranges of 110°–140° and 220°–250° during planning.

The median total processing time from exporting the plan from Monaco to performing gamma analysis in Eclipse was 3 min, ranging from 2 to 5 min.

## Discussion

In this study, we explored the use and ease of integration of AAA-based Eclipse TPS for independent MU QA of online adapted plans generated on the Elekta Unity MR-Linac system using Monaco TPS. Monaco beam characteristics were modified to allow plan import into Eclipse. Our study showed that the AAA-based dose calculation in Eclipse TPS can be used as a secondary dose calculation QA to verify a plan generated in the presence of a magnetic field. The gamma comparisons are >95% using a 3%/3 mm gamma pass rate criterion. A few individual beams have a pass rate below 90%. The gamma comparisons are >92% using a 3%/2-mm pass rate criterion. More plans have one beam with the individual beam pass rate below 90%. For clinical use, we chose the 3%/3 mm gamma pass rate criterion. The cause for the low pass rate of these beams is discussed below.

In addition to the calculation comparison, the IMU analysis was compared with the measurement-based analysis. The statistically significant contingency correlation analysis validated the ability of IMU analysis tracking measurement-based system even in the absence of the B-field. In addition, the IMU also demonstrated the ability to perform a 2D comparison with Monaco TPS using a reasonable gamma criterion (3%/3 mm), providing a significant improvement over the single point verification technique (8) offered by the current commercial system.

However, the inaccurate couch modeling in the Monaco TPS attributed certain ArcCheck failure at certain gantry angle ranges. As the IMU has the same couch information as Monaco TPS, it is currently able to detect the couch transmission deficiency resulting in a false negative. A more sophisticated couch model should be investigated and implemented in Monaco TPS to improve both the ArcCheck and IMU in the future.

The AAA algorithm calculates dose without including the effect of a magnetic field. In our comparison, the B-field effect was partly included by shifting the plane where gamma analysis is performed. Larger differences can still be seen near tissue–air interfaces due to the electron return effect. The default plane is the plane perpendicular to the beam axis through the default reference point in the plan. This reference point is usually the isocenter in Monaco. Because the couch tabletop is fixed on Unity, the isocenter could potentially be outside the target. A user can also use the center of the mass of the planning target volume (PTV) to shift the plane through it or choose another point to avoid the air–tissue interface. Another tool available to choose is to define a customized region of interest (ROI) that can be used to exclude the air cavity if the beam CIAO area is affected by it. The results for one head and neck plan are shown in **Table 2**. Because of the airway, beam 12 has a very low pass rate of 69.2% with the beam CIAO. With the airway removed from the CIAO, the pass rate is improved to 81.8%. The average pass rate also improved from 89.9% to 93.8%. The effect of a magnetic field at tissue–air or tissue–lung geometry interface results in dose enhancement at the interface due to the electron return effect. ERE is very challenging to incorporate into model-based dose calculation algorithms that are currently being used for independent dose checks. Hence, larger interface discrepancy will be seen in anatomical locations with significant heterogeneities such as HN and the lung. The cases analyzed in this study included the pancreas and pelvis. For heterogeneous geometries, we will need to develop additional tools to deal with the situation where lung tumors are surrounded by large air cavities and the planar dose distributions may be significantly affected by the electron return effect. A recent study has looked at replacing two-dimensional gamma analysis with three-dimensional-based analysis, such as three-dimensional gamma and dose–volume histogram (DVH) comparisons (21) to provide a more realistic comparison with the planning criteria as also recommended by recently published TG219 (9).

**Table 2:** Gamma pass rate for a plan with and without airway removed from the ROI.

Beam	Regular ROI	ROI with airway removed
1	93.6	97.5
2	92.9	95.0
3	90.1	94.4
4	93.3	96.8
5	87.3	93.3
6	90.4	96.9
7	91.3	97.6
8	96.0	98.5
9	96.5	94.6
10	92.0	90.6
11	93.7	88.0
12	69.2	81.8
13	82.1	93.8
Average	89.9	93.8

ROI, region of interest.

We used planar dose for gamma analysis, which can be more sensitive to the magnetic field effect and the dose gradient in the plane normal direction. In this study, we only explored 2D gamma analysis, but since Eclipse calculation is based on the 3D patient volume, one can also perform a 3D gamma analysis for the plan. We are currently exploring if the 3D gamma analysis can be performed within a reasonable time frame of online adaptive planning QA. 3D gamma analysis will also be less sensitive to the presence of any air cavities in the proximity of the target and the high dose gradient in the plane normal direction.

The independent MU workflow including plan export, plan conversion, 3D dose calculation, and gamma analysis usually takes about 3.2 ± 0.9 min. The bulk of the time is spent in Eclipse dose calculation within the 3D heterogeneous patient geometry. We use an Eclipse configuration that combines Citrix application virtualization and a distributed calculation framework that includes 18 Eclipse fast calculation servers. The MR 3D image is assigned a voxel-wise electron density for dose calculation. The total number of voxels and the number of beams can affect the dose calculation speed, as Eclipse needs to preprocess the electron density for each voxel for the AAA dose calculation. Reducing the number of structures to export from Monaco can help further reduce the calculation time if the MR size is large and has a finer image resolution of 1 mm or less. A more powerful dedicated front-end Eclipse workstation can also help, as the virtual Citrix server has limited resources to process the image more efficiently before the data are passed to the fast calculation servers. Compared to other existing approaches that use point dose comparison, we are performing a full 3D dose calculation in patient geometry with heterogeneity correction. The IMU task is performed in parallel to the other online planning activities such as plan evaluation and approval by the physician to improve efficiency.

We should point out that the modified plans stored in Eclipse do not pose any clinical safety issues. First, the plans in the Elekta Monaco system are not affected in any way by this process. Their records are kept in a different database. Second, those QA plans are assigned a special machine in Eclipse for dose calculation and independent MU check only, which is not connected to any physical treatment machine and is thus undeliverable. These plans are thus identified as Monaco QA plans in case they are retrospectively examined or audited in the future. Third, the lateral shift of 2 mm to account for the B-field effect is only applied during the gamma analysis step when the gamma analysis is performed. It does not change the state of the dose stored in the Eclipse system.

Although our IMU workflow is specifically developed for online adaptive planning for a 1.5-Tesla MR-Linac system, it can also be applied to perform independent QA of offline reference plans with CT or MR images. We routinely use it for our reference plan IMU QA and have been obtaining similar results. The current IMU workflow and implementation can also be extended to another hybrid MR-Linac system with low magnetic field strengths where the impact of ERE would be minimal.

## Conclusion

The Eclipse TPS provides an efficient and streamlined way to perform independent dose calculation QA for online adaptive planning on the Elekta Unity MR-Linac system. Future work will include 3D gamma analysis using Eclipse IMU to further minimize the effect of the magnetic field.

## Data Availability Statement

The raw data supporting the conclusions of this article will be made available by the authors, without undue reservation.

## Author Contributions

SL developed the conception of the project, developed the Eclipse beam model, and made dosimetry measurements. JY developed the in-house software and wrote the manuscript. PZ coordinated all developments. NT oversaw the development on the Monaco side. She collected and analyzed data with PS, ES, and JL. DL oversaw the dosimetry development. JM oversaw and approved the design and use. AL oversaw the software development. All authors reviewed and revised the manuscript.

## Funding

This research was partially supported by the NIH/NCI Cancer Center Support Grant/Core Grant (P30CA008748).

## Conflict of Interest

The authors declare that the research was conducted in the absence of any commercial or financial relationships that could be construed as a potential conflict of interest.

## Publisher’s Note

All claims expressed in this article are solely those of the authors and do not necessarily represent those of their affiliated organizations, or those of the publisher, the editors and the reviewers. Any product that may be evaluated in this article, or claim that may be made by its manufacturer, is not guaranteed or endorsed by the publisher.
